# Three-photon imaging of hippocampal neurogenesis through the intact mouse brain

**DOI:** 10.21203/rs.3.rs-8267806/v1

**Published:** 2025-12-19

**Authors:** Yusaku Hontani, Xin Su, Yicheng Wu, Diana Machado, Megumi Mizoguchi, John Darby Cole, Hansjörg Kasper, Philipp Bethge, Fritjof Helmchen, Sebastian Jessberger

**Affiliations:** 1 Laboratory of Neural Plasticity, Faculties of Medicine and Science, Brain Research Institute, University of Zurich, Zurich, Switzerland.; 2 Current address: Department of Neuroscience and Mahoney Institute for Neurosciences, Perelman School of Medicine, University of Pennsylvania, Philadelphia, PA, USA.; 3 Neuroscience Center Zurich, University of Zurich and ETH Zurich, Zurich, Switzerland.; 4 Laboratory of Neural Circuit Dynamics, Faculties of Medicine and Science, Brain Research Institute, University of Zurich, Zurich, Switzerland.; 5 University Research Priority Program (URPP), Adaptive Brain Circuits in Development and Learning (AdaBD), University of Zurich, Zurich, Switzerland.

## Abstract

Multiphoton imaging allows for the visualization of structural and functional plasticity within the central nervous system. However, gaining optical access to deep brain structures, such as the hippocampal dentate gyrus (DG), requires invasive approaches, causing brain damage. Here we optimize three-photon (3P) microscopy to perform longitudinal imaging of the DG in the intact brain at unprecedented depth of up to 1800 μm. We apply this approach to follow the dynamics of neural stem cells (NSCs) in the adult and developing DG, allowing for novel insights into structural plasticity deep within the intact mouse brain.

Multiphoton imaging is used extensively to study dynamic cellular processes in various tissues of mammals, including the rodent brain^1^. While two-photon (2P) microscopy allowed for fundamentally novel insights into brain function and plasticity, optical access to the adult mouse brain beyond 700 μm in depth requires the removal or damage of overlying tissues for high-contrast cellular imaging^1^. For example, parts of the cortex need to be removed to study neural stem cell (NSC) dynamics by 2P imaging in the dentate gyrus (DG) of the mouse hippocampus, a brain region that is permissive of lifelong neurogenesis and that plays a critical role in certain forms of learning and memory^2–5^. The development of three-photon (3P) microscopy expanded the reachable imaging depth^6^. While 3P imaging achieves high-contrast cellular imaging down to ~1700 μm in prefrontal cortex of the intact mouse brain^6, 7^, access to the hippocampus is still limited to approximately 1400 μm for cellular imaging. This is primarily due to the need to pass through the corpus callosum, a highly light-scattering structure due to myelination^6, 8^. Therefore, *in vivo* cellular imaging in many areas within the intact brain, including the DG, is limited (Fig. 1a).

To overcome currently existing imaging depth limitations, we calculated the effective 3P excitation spectrum by combining 3P excitation cross sections, maximum permissive laser power, available pulse energy under the objective, and laser attenuation lengths (Fig. 1b–c). We found that the effective 3P excitation efficiency of the red fluorescent protein tdTomato peaks at ~1650 nm wavelength for a depth of 1600 μm in the mouse brain, required to reach the DG (Fig. 1c). To avoid potential tissue damage that could occur with long-term imaging, we reduced the pulse repetition rate to 250 kHz, allowing for high pulse energy (maximum ~200 nJ under the objective) while maintaining relatively low average laser power (maximum ~50 mW) at the brain surface. We tested this approach by labeling neural stem cells (NSCs) and their progeny in the DG with a red-shifted fluorophore using Gli1-Cre^ERT2^:tdTomato transgenic mice^9^. While the area of the skull overlying the hippocampus was removed and covered with a glass window (Extended Data Fig. 1a–b) the brain itself remained intact. Two weeks after implantation of a cranial window, tamoxifen (TAM) was injected to induce genetic labeling with tdTomato of NSCs and their daughter cells. Strikingly, we identified high-contrast fluorescence signal within the DG upon TAM-mediated cell labeling at depths of up to 1800 μm, substantially exceeding previous depth limits using genetically labeled cells in the hippocampus of the intact mouse brain analyzed by 3P microscopy (Fig. 1d, Extended Data Fig. 1c, and Supplementary Video 1)^10^.

After establishing a 3P-based approach to detect genetically encoded fluorescence signals beyond previously obtained depths, we next aimed to probe for the feasibility of chronic imaging within the same region of interest. We used genetic labeling of NSCs to analyze the dynamics of adult hippocampal neurogenesis on the time scale of weeks and months, which was previously only possible through 2P imaging with invasive removal of overlying cortex or even the hippocampal subregion CA1^11^. Two weeks after cranial window surgery we injected TAM and started imaging 4–12 days later for up to 86 days in the intact brain (Fig. 1e and Extended Data Fig. 1d), achieving high-contrast visualization of NSCs and newborn neurons in the DG (Supplementary Video 2). To further improve image quality, we applied a deep learning-based denoising approach using Noise2Void (Fig. 1e and Extended Data Fig. 1e–f)^12^. Denoising of acquired images across different timepoints facilitated *post hoc* lineage tracing of genetically labeled NSCs and their progeny (Fig. 1f and Extended Data Fig. 2).

We compared key metrics of NSC behavior with the 3P-based intact brain preparation obtained here with an existing dataset derived from 2P imaging that required cortex removal^9^. We measured duration until first radial glia-like (R) cell division, duration between consecutive R cell divisions, clonal output (surviving cells generated from a single NSC), survival rate of newborn cells, and time until death for newborn cells (Fig. 1g–k). These parameters showed no significant differences in NSC/progeny behavior between 3P versus 2P approach (intact brain versus cortex removed). However, removal of cortical areas can affect the animals’ physiology and behavior, even though these consequences may escape rather insensitive behavioral tasks^13^. Notably, the 3P approach resulted in laser pulse energy of only ~1.3–1.6 nJ at the beam focus, making nonlinear damage highly unlikely^14, 15^. Consistently, we did not detect any signs of thermal damage comparing expression of Iba1-labeled microglia and GFAP/SOX2-labeled astrocytes between ipsilateral (imaged) and contralateral (control) hemispheres within the same animals using immunohistochemical analyses (Extended Data Fig. 3a–l)^16^. Chronic 3P imaging also did not alter NSC dynamics, as indicated by comparable numbers of doublecortin (DCX)-expressing (newborn) and Ki67-labeled (dividing) cells in the DG (Extended Data Fig. 4a–f), again comparing imaged (ipsilateral) versus non-imaged (contralateral) hippocampi. Thus, the 3P approach applied here is capable to track individual cells (here NSCs and their progeny) at unprecedented depth over extended time periods in the intact adult mouse brain.

A large portion of the DG is generated between postnatal days (P) 5 to 8^17, 18^. However, this key developmental stage so far remained inaccessible for chronic imaging, since the DG in early postnatal mice is beyond 2P imaging depth and any surgical removal of overlying brain tissues is not feasible in mice at this age. This is in contrast to cortical neurons that can be tracked by 2P imaging at comparable developmental stages over time^19^. Here, we aimed to test the 3P imaging approach in the developing DG of early postnatal mice (P5–8) (Fig. 2a–b). Strikingly, chronic intravital 3P imaging was capable of successfully capturing tdTomato-labeled NSCs and their daughter cells with high imaging contrast, along with third-harmonic generation (THG) signals, at a depth of approximately 1100 μm (Fig. 2c). Notably, the same region of the DG could be traced over consecutive days (Fig. 2d–e), revealing the addition of new cells and showing the feasibility to visualize NSC dynamics in the early postnatal DG. Thus, the set-up described here will allow for future experiments that will define the hitherto poorly understood cellular principles underlying early postnatal DG neurogenesis and the subsequent establishment of the adult neurogenic niche^20^.

We here report a long-wavelength, chronic 3P imaging approach that achieves high-resolution cellular imaging at unprecedented depths in the intact mouse brain, overcoming the *in vivo* imaging depth limit of genetically labeled cells within brain tissues^1, 10^. Together with methods enabling multicolor 3P imaging that, for example, will allow for simultaneous imaging of distinct cell types (e.g., microglial or endothelial cells) within the same area, the approach presented here expands the toolbox to study structural plasticity at great depth in intact tissues^10, 21, 22^. Optimized 3P imaging will become a new standard for deep imaging, which will open novel avenues for studying cellular dynamics in complex tissues, such as the developing and adult mammalian brain.

## Online Methods

### Chronic cranial window implantation in adult mice

All animal experiments were approved by the Cantonal Commission for Animal Experimentation of the Canton of Zurich (ZH130/23, ZH019/24), Switzerland. We used mixed sex *Gli1-Cre*^*ERT2*^*;tdTomato* mice (*Gli1-Cre*^*+/−*^*Ai14*^*+/+*^), generated by crossing *Gli1-Cre*^*ERT2*^ mice (Gli1^tm3(cre/ERT2)Alj^; The Jackson Laboratory, 007913) and CAG tdTomato (Ai14; B6.Cg-Gt(ROSA)^26Sortm14(CAG-tdTomato)Hze^; The Jackson Laboratory, 007914) reporter strain, as reported previously^9^. 8–12-week-old mice underwent surgery where the cranial bone was circularly removed (diameter: 5 mm) with a dental drill^9^. A glass window (5.0 mm diameter, 72296–05, Electron Microscopy Sciences) was pre-attached to a 0.5 mm high steel cannula (outer diameter: 5.0 mm, inner diameter: 4.0–4.5 mm) using a UV-light curing adhesive (NOA81, Norland Products). This unit was placed on the open brain tissue and secured by attaching the rim of the cannula to the surrounding skull using blue light curing dental cement (Tetric EvoFlow T, Ivoclar Vivadent) and centered above the dorsal dentate gyrus (DG) (+2.0 mm posterior, +2.5 mm lateral from bregma). The exposed skull was treated with an etching material (iBOND Total Etch, Kulzer). A self-made metal headpost (0.6–1.0 g weight) was then implanted onto the skull using the blue-light curing dental cement. Two weeks after the craniotomy, tamoxifen (TAM, solubilized in 90% corn oil and 10% ethanol) was administered via intraperitoneal (i.p.) injection at a concentration of 70–130 mg/kg to achieve sparse labeling of genetically targeted R cells. In total eleven mice were used for experiments, with three mice included here that showed sufficient fluorescent signal and window quality allowing for longitudinal imaging over weeks/months. The remaining eight mice either did not show clear fluorescence signals or deteriorating window quality across imaging data points and were excluded from data collection.

### Chronic cranial window implantation in neonatal mice

We used *Gli1-Cre*^*ERT2*^*;tdTomato* mice (*Gli1-Cre*^*+/−*^*Ai14*^*+/+*^) for three-photon (3P) imaging in neonates. Mice received TAM via i.p. injection at P6 (70 mg/kg) for Fig. 2c and at P4 (30 mg/kg) for Fig. 2d–e. 24 hours later, anesthesia was induced using 5% isoflurane mixed with oxygen for 5 minutes. Mice were then transferred to a custom-made stereotaxic stage and placed on a heating pad to maintain the body temperature at ~37°C. Anesthesia was subsequently maintained via a mask using 2% isoflurane mixed with oxygen. The skin was disinfected using 70% ethanol. A topical anesthetic (Emla cream) was applied to the surgical site. Following the removal of the scalp using dissection scissors, a local anesthetic splash block consisting of 10 mg/kg lidocaine and 2 mg/kg bupivacaine was applied. The craniotomy (~3 mm diameter) was performed with the tip of a 26-gauge injection needle (Sterican, Braun). The center of the craniotomy was located ~1.5 mm laterally from the midline and at the middle point between the bregma and lambda for the anterior/posterior coordinate. A glass window with 5 mm diameter (72296–05, Electron Microscopy Sciences) was attached to the skull to cover the open brain tissue using dental cement (C&B Metabond, Parkell). A 3D-printed plastic headpost (~0.1 g weight) was secured on top of the glass window using blue-light curing dental cement (Tetric EvoFlow T, Ivoclar Vivadent). In total six mice were used for early DG imaging experiments, with one mouse included here that showed sufficient fluorescent signal and window quality allowing for longitudinal imaging. The remaining five mice either did not show clear fluorescence signals or deteriorating window quality across imaging data points and were excluded from data collection.

### Calculation of effective three-photon excitation spectrum

The effective 3P excitation spectrum of tdTomato at 1600 μm depth in the adult mouse brain was calculated assuming a Gaussian beam focus. The 3P signal generated per second is defined by the following equation^16, 23^:

(1) $$S_{3\text{P}}=\frac{1}{3}\frac{g_{p}^{\left(3\right)}}{{\left(f\tau \right)}^{2}}\phi \eta \sigma_{3}Cn_{0}\frac{2\pi^{2}{\left(NA\right)}^{2}{\langle P\left(t\right)\rangle }^{3}\text{exp}\left(-3\times \text{EALs}\right)}{3\lambda^{3}}$$

where *f* is the laser repetition rate, *τ* is the laser pulse duration, *g*_p_^(3)^ is the 3rd-order temporal coherence of the excitation source, *ϕ* is the system collection efficiency, *η* is the fluorescence quantum efficiency, *σ*_3_ is the 3P excitation cross section, *C* is the fluorophore concentration, *n*_0_ is the reflective index of the medium (*i.e*., water), *λ* is the 3P excitation wavelength, *NA* is the numerical aperture of the objective lens, 〈*P*(*t*)〉 is the photon flux at the tissue surface (photons/s), *EALs* is the effective attenuation lengths. 1 EAL is equivalent to the length where the pulse energy is attenuated to 1/e. We assumed that the pulse duration (*τ*), refractive index (*n*_0_), and *NA* are constant across different excitation wavelengths. *g*_p_^(3)^, *ϕ, C* are not wavelength dependent. Since the photon flux per second 〈*P*(*t*)〉 ∝ *fI* / *λ*, where *I* is the pulse energy at the tissue surface, the wavelength-dependent parameters are *ησ*_3_, *f*, *I*, and *EALs*. Therefore, Eq. (1) can be simplified to:

(2) $$S_{3\text{P}}\propto \eta \sigma_{3}fI^{3}\text{exp}\left(-3\times \text{EALs}\right)$$

**Figure 1.**
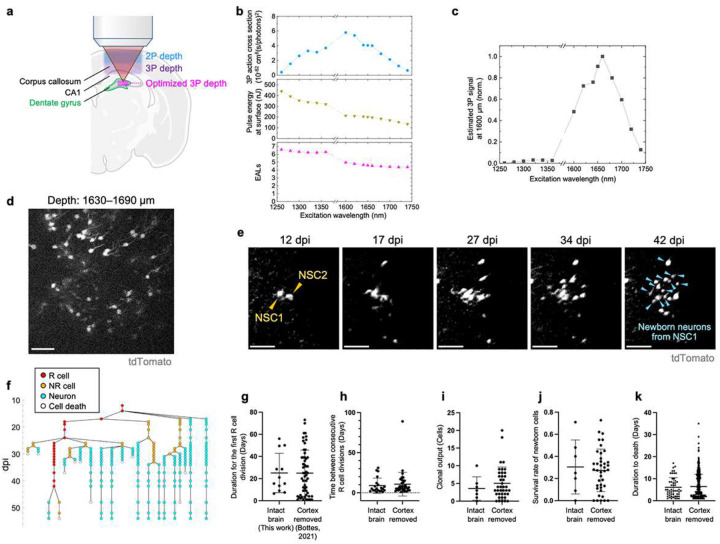
Long-term three-photon (3P) imaging to trace neural stem cell (NSC) dynamics in the intact, adult mouse brain. **a**, Schematic of multiphoton imaging in the intact, adult mouse brain, showing typical cellular imaging depth limit of two-photon (2P) and 3P microscopy, highlighted by the blue and purple squares, respectively. The imaging region of this study in the dentate gyrus (DG) is indicated by the magenta square. **b**, Wavelength dependence of 3P action cross sections of tdTomato^22, 24^ (blue), estimated pulse energy under the objective of the excitation laser (dark yellow), and effective attenuation lengths (EALs) at 1600 μm depth (magenta). **c,** The calculated effective 3P excitation spectrum of tdTomato at 1600 μm imaging depth. **d**, A snapshot 3P image in the adult mouse hippocampus at a depth of 1630–1690 μm at 65 days post-tamoxifen (TAM) induction (dpi). **e**, Selected images from longitudinal 3P imaging. Orange arrowheads at 12 dpi indicate two NSCs (NSC1 and NSC2), and the blue arrowheads at 42 dpi show newborn neurons derived from NSC1. **f**, A lineage tracing of NSC1, indicated in panel e. For lineage tree of NSC2 refer to lineage 2 in Extended Data Fig. 2. **g-k**, Comparison of NSC dynamics between intact and cortex-removed brains. Left and right columns show data from 3P imaging in the intact brain (this work, analyzed from Extended Data Fig. 2) and 2P imaging in cortex-removed brain reported previously^9^, respectively. Duration of the first R cell division (g), days between consecutive R cell divisions (h), number of newborn neurons generated from a single NSC (i), survival rate of newborn cells (j), and duration to death of newborn cells (k). *p* values: 0.99 (g), 0.56 (h), 0.34 (i), 0.76 (j), and 0.55 (k). Scale bars represent 50 μm.

3P action cross sections *ησ*_3_ were collected from reported literature for the 1300-nm window (1260–1360 nm)^22^ and the 1700-nm window (1600–1740 nm)^24^, normalized using the value at 1650 nm between the two literatures. (Fig. 1b, top panel). The laser pulse energy *I* was the measured pulse energy under the objective of our system (Fig. 1b, middle panel). At 1600 μm, the total EALs were calculated as summation of the EALs from the cortex and the corpus callosum, which have a distinct EAL. We estimated the thickness of cortex and corpus callosum as 1400 μm and 200 μm, respectively. The EAL for the cortex was based on reported values calculated by scattering theory and water absorption, assuming 75% water composition^6, 25^ (Fig. 1b, bottom panel). The EAL for the corpus callosum was assumed to be 120 μm and 150 μm for the 1300-nm and 1700-nm windows, respectively^6, 16, 26^. Note that tissue aberration was not considered in the calculation. The laser repetition rate *f* was calculated based on the maximum permissive laser power (*P*_max_) to avoid tissue damage, where *f* = *P*_max_ / *I*. *P*_max_ was assumed to be 100 mW for the 1300-nm window^16^ and 50 mW for the 1700-nm window. For example, at 1650 nm, *P*_max_ = 50 mW and *I* = 200 nJ, then *f* = 50 mW / 200 nJ = 250 kHz. The resulting effective 3P excitation spectrum at 1600 μm depth based on Eq. (2) is shown in Fig. 1c.

### Three-photon microscopy setup

3P excited fluorescence images were acquired with a multiphoton microscope (VivoScope, Scientifica) that has two detection channels with GaAsP photomultiplier tubes (PMTs) (H11706P-40, Hamamatsu Photonics). For 3P excitation, the output from an optical parametric amplifier (OPA) (Mango, APE) was used, centered at 1650 nm. The OPA was pumped by a 50 μJ, 1030-nm laser operating at 250 kHz (Satsuma HP3, Amplitude), providing a maximum output of 800 nJ. The laser pulse dispersion introduced by the microscope optics was pre-compensated by perpendicularly transmitting the beam through a 2-mm-thick Si wafer with an anti-reflection coating (17–202, Edmund Optics). The pulse duration under the objective lens was 65 fs (full width at half maximum, assuming a sech^2^ temporal profile). The pulse energy and average power used for *in vivo* DG imaging were ~160–200 nJ and ~40–50 mW, respectively, at the brain surface. After passing through a custom-ordered primary dichroic mirror (cutoff: 700 nm), the laser was projected to an objective lens (XLPLN25XSVMP2, Olympus, numerical aperture (NA) = 1.0, 4 mm working distance). The objective was used with D_2_O immersion medium to minimize excitation laser absorption by H_2_O at the 1650 nm wavelength. The objective back aperture (aperture size: 14.4 mm) was underfilled by the excitation beam (diameter: ~11 mm at 1/e^2^). The effective NA was consequently estimated to be approximately 0.75. Fluorescence from tdTomato and the third-harmonic generation (THG) signal were epi-collected through the objective lens. The excitation laser was blocked by a near-infrared block filter (TF1, Thorlabs). The signal was then separated into two channels by a dichroic mirror with a 565 nm cut-off (T565lpxr, Chroma). The signals were detected by the GaAsP PMTs after passing through the following interference filters: 520 ± 30 nm (FF01–520/60, Semrock) for THG signal and 607 ± 35 nm (FF01–607/70, Semrock) for tdTomato fluorescence.

### Three-photon in vivo imaging

**Extended Data Figure 1.**
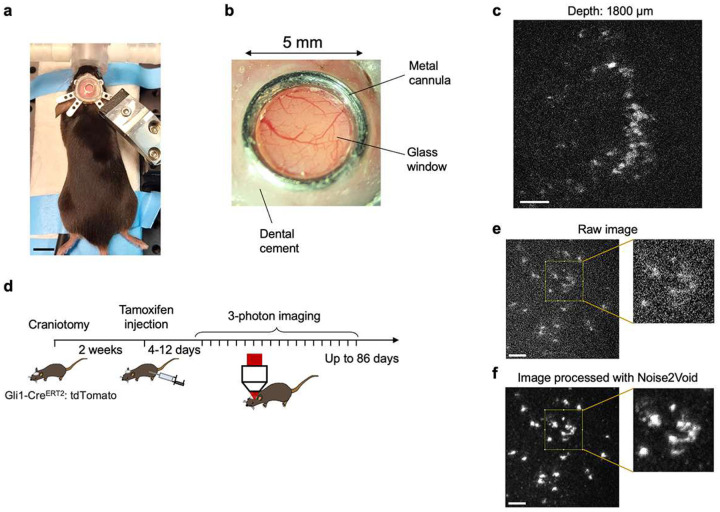
Longitudinal 3P *in vivo* imaging. **a,** Picture of an adult mouse with a metal headpost for 3P imaging. **b**, Close-up view of the cranial window. The glass window attached with a metal cannula was implanted to cover the brain tissue after skull removal. **c**, 3P *in vivo* images obtained at a depth of 1800 μm below the brain surface. **d**, Timeline of the longitudinal 3P imaging experiments. TAM was injected 2 weeks after craniotomy to achieve sparse labeling of NSCs. 3P imaging was initiated 4–12 days after TAM induction and continued up to 86 days. **e-f**, Denoising with Noise2Void. Raw 3P image obtained in the adult DG (e) and denoised image using Noise2Void (f). Boxed areas are shown in higher magnification. Scale bars represent 1 cm (a) and 50 μm (c, e-f).

**Figure 2.**
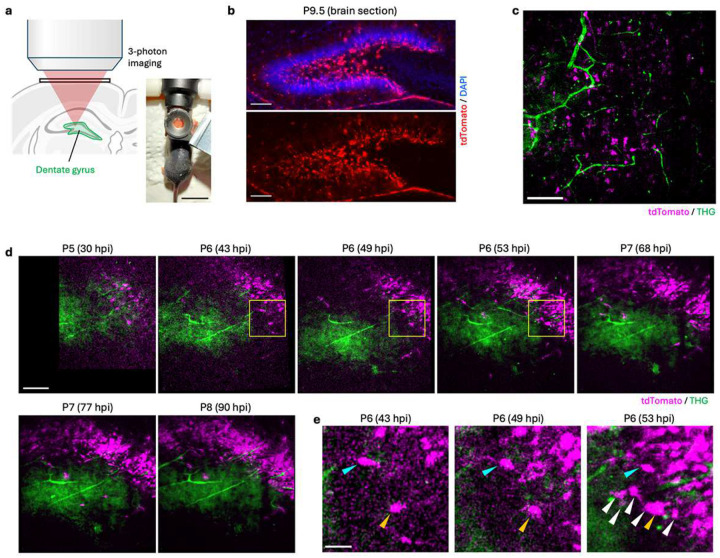
*In vivo* 3P imaging of early-postnatal dentate gyrus. **a**, Schematic of 3P imaging in the early-postnatal mouse brain. The picture shows a neonatal mouse with a 3D-printed plastic headpost. **b**, A widefield fluorescence image of a brain slice from a postnatal day (P) 9.5 Gli1-tdTomato mouse, 4 days after TAM administration. Blue: DAPI, Red: tdTomato. Lower panel shows tdTomato-only signal. **c**, A snapshot of an *in vivo* 3P image obtained at P7, 1 day post-TAM induction at 1030–1130 μm depth from the brain surface. **d**, Longitudinal *in vivo* 3P images acquired at ~1100 μm depth over 90 hours post-TAM induction (hpi). **e**, Close-up images from selected regions outlined by the yellow boxes in d. Blue arrowhead points toward a migrating cell. Orange arrowhead points towards a cell that appears to undergo cell division. Note the appearance of numerous newborn cells (white arrowheads). Green: third-harmonic generation (THG), magenta: tdTomato-labeled cells. Scale bars represent 1 cm (a), 100 μm (b-d), and 30 μm (e).

Anesthesia was maintained via a mask supplying 1–2% isoflurane mixed with oxygen on a heating pad, which was used to maintain the mouse’s body temperature at ~37°C. The headpost was secured with a metal clamp to a custom stage to suppress motion. The cranial window was cleaned with deionized water and a surgical sponge (Sugi Sponge Points, Questalpha) to optimize optical clarity before placing the mouse under the objective. The mouse breathing was closely monitored with a CMOS camera (CS165MU, Thorlabs) together with NIR LED (M970L4, Thorlabs) illumination, and the breathing frequency was adjusted to ~1 Hz by controlling the isoflurane level during imaging. 3P images were obtained using the following settings: pixel size: 512 × 512 pixels, the pixel dwell time: 4 μs, a frame rate: 0.86 s, the depth increment for Z-stack images: 3 μm. The field-of-view (FOV) was set at 324 × 324 μm^2^ (for Fig. 1d, Extended Data Figs. 1c,e–f and 5) or 360 × 360 μm^2^ (for Fig. 1e) for adult mice and 540 × 540 μm^2^ for neonatal mice. The number of frames obtained for averaging differed depending on the image quality, typically ranging between 40 and 80 frames for adult mice, with the exception of 500 frames averaged at 1800 μm imaging depth (Extended Data Fig. 1c). For neonates, 20 frames were averaged for each depth. To prevent the gradual replacement of the D_2_O immersion medium by H_2_O from the atmosphere, which causes significant excitation laser absorption at 1650 nm, the D_2_O medium was replaced every 15 minutes during the imaging session. Longitudinal imaging was performed with a maximum frequency of once per day for adult mice: at 12–54 dpi (Mouse #1), 4–86 dpi (Mouse #2), and 5–82 dpi (Mouse #3). For the neonatal mouse, longitudinal 3P imaging was performed at 30, 43, 49, 53, 68, 77, and 90 hours after TAM induction (hpi) (Fig. 2d–e). To ensure accurate imaging of the same FOV across multiple longitudinal sessions, a precise two-step optimization relying on cellular landmarks was used. First, the edge of the implanted glass window was first located. Then, the stage was moved laterally and axially by previously recorded values, which allowed for a coarse positioning to the target FOV. Finally, the correct FOV was identified by finding the FOV that best matched the unique three-dimensional pattern of fluorescently labeled cells and/or THG within the target area.

### Hand feeding of neonatal mice

Following the headpost implantation surgery, the neonatal mouse (starting at P5) was provided with nutritional support through hand feeding every two hours, as previously reported^27^. The hand feeding regimen was performed over a short period, lasting from P5 until P8, corresponding to the end of the longitudinal imaging period. For a single feeding, ~200–300 μl of vet milk was warmed to 37°C. Then, the milk was administered using a painting brush while gently holding the mouse by hand. During feeding, the body of the mouse was gently massaged to ensure smooth milk consumption and to mitigate the risk of choking. After each feeding session, the mouse was immediately returned to the home cage. The cage was placed on a heating pad, maintaining the ambient temperature around 30°C until the next scheduled feeding session. Strict termination criteria (e.g., weight loss of >10% relative to control mice) resulted in survival of approximately 70% of mice undergoing surgery.

### Photobleaching analysis

Photobleaching of the fluorescence signal was analyzed in 3P imaging at a depth of ~1600 μm in the hippocampus of an adult mouse (Extended Data Fig. 5a–b). The analysis was performed using the maximum *in vivo* imaging setting: 50 mW average power (200 nJ pulse energy) at the tissue surface. 9 regions of interest (ROIs) were selected from the same FOV. The mean fluorescence intensity in each ROI was continuously calculated over 30 time frames, equivalent to a total duration of 34.9 s. The decay of the mean fluorescence intensity was fit with an exponential decay function: *F(t) = F*_0_ *exp(−t/τ*_d_). The decay time constant *τ*_d_ calculated from the fit was found to be >10^10^ s. This large time constant quantitatively indicated that photobleaching was not reasonably detected within the 34.9 s detection time under these imaging conditions.

### Image processing

For all longitudinal Z-stacks, a selected depth range of ~20–30 μm (corresponding to the subgranular zone and the bottom part of the granule cell layer) was projected as maximum intensity profiles. The same depth range was used for the same animal. The maximum-projected images from different imaging sessions were then stacked together to generate a time-lapse movie. The time-lapse movie was processed with the MultiStackReg plugin in ImageJ software (applying a rigid body transformation) to align the ROIs laterally across time points and truncated to select the ROI. Prior to performing lineage tracing for adult brain imaging, denoising was conducted using the Noise2Void (N2V) self-supervised framework (Fig. 1e and Extended Data Fig. 1e–f). The N2V model was trained using 155 distinct images and validated on a separate set of 40 images, all of which were acquired from the adult DG, totaling 195 images used. The network architecture utilized was a standard U-Net with a convolution kernel size of 3. Batch normalization was applied throughout the network. The optimization process minimized the mean squared error (MSE) loss function, with a training batch size of 64. Training was executed for 20 steps per epoch. Specific to the N2V self-supervised strategy, training patches were set to a shape of (128, 128). The proportion of pixels masked and excluded from the prediction target (N2V perc pix) was set to 0.198. The context for the prediction was defined by an N2V neighborhood radius of 5 pixels, and the masked pixel replacement strategy (N2V manipulator) was uniform_withCP. The resulting trained N2V model was subsequently applied to all longitudinal images used for lineage tracing within the adult DG (seven regions of interest from three mice).

### Lineage tracing and NSC dynamics analysis for adult mice

**Extended Data Figure 2.**
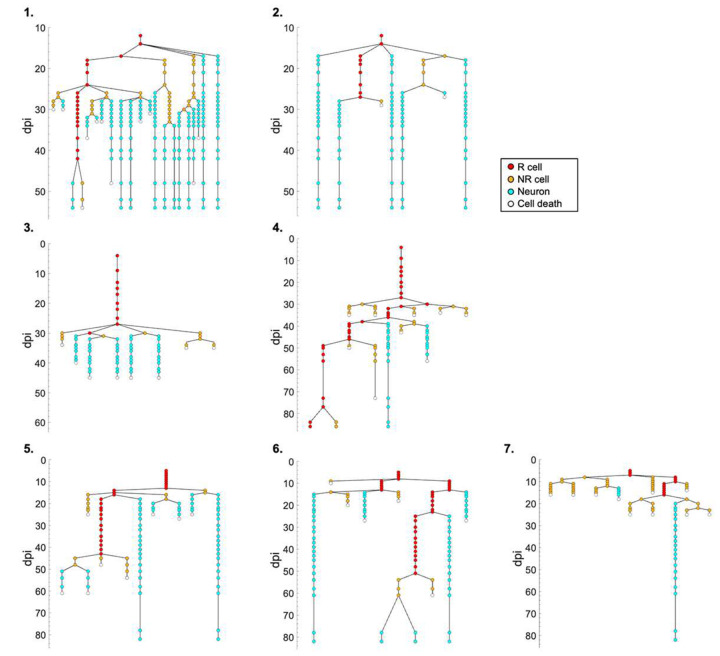
Lineage trees of NSCs in the adult DG. Lineage traces of 7 NSCs from 3 mice in the adult DG. Red, orange, and cyan circles indicate radial glia-like (R) cells, non-radial glia-like (NR) cells, and neurons, respectively. White circles represent cell death. Note: lineage 1 is also shown in Fig. 1f.

Seven NSC clones from three adult mice were used for lineage tracing (Mouse #1 (male): Fig. 1f and panel 1–2 of Extended Data Fig. 2, Mouse #2 (male): panel 3–4 of Extended Data Fig. 2, and Mouse #3 (female): panel 5–7 of Extended Data Fig. 2). Lineage tracing was performed using time-lapse images obtained as described above. Identification of R cells, NR cells, and neurons was performed as described before^9^. Cell death was defined as a cellular disappearance (irreversible loss of fluorescence signal), specifically confirmed not to be due to cell migration outside the FOV. Cells that migrated outside the FOV were rare and excluded from the lineage analysis.

The following five parameters were extracted to quantify the dynamics of NSCs and their daughter cells (Fig. 1g–k):

1. Duration to the first R cell division after TAM induction.
2. Time between consecutive R cell divisions.
3. Clonal output, i.e., the number of surviving cells derived from a single R cell.
4. Survival rate of newborn cells derived from a single R cell.
5. Duration to death of newborn cells after the birth of the cell.

To accurately quantify time-dependent events related to parameters (1), (2), and (5), the date of the event was defined as the midpoint between the time point the event was observed and the time point immediately preceding the observation. For example, if cell death was observed at 60 dpi (and the preceding time point was 58 dpi), the cell death date was defined as 59 dpi. Similarly, a cell birth detected between 15 and 16 dpi was assigned an event date of 15.5 dpi. To compare our results obtained with 3P imaging in the intact brain with 2P imaging studies previously reported in cortex-removed brains^9^, we reanalyzed the NSC dynamics using all displayed lineage trees. Note that the same transgenic mouse line, age range, and recovery duration after surgery were applied in the previous study. For the analysis of parameters (3) and (4), only the complete lineage trees were considered from the previous study, defined as those having at least 5 days after the final cell division.

### Immunohistochemistry

After final 3P imaging sessions, adult mice were first anesthetized via i.p. injection of a lethal dose of pentobarbital and then transcardially perfused with cold saline, followed by 4% paraformaldehyde (PFA). Brains were postfixed overnight in 4% PFA at 4 °C. Brains were transferred to 15% sucrose solution for 6–8 hours, then soaked in 30% sucrose for more than 24 hours for cryoprotection. The brain tissue was coronally sectioned using a sliding microtome (SM2010R, Leica) with a thickness of 40 μm along the entire extent of the DG.

Every sixth section (covering the entire DG) were used for immunohistochemistry, as described before^9^. Primary antibodies used: anti-tdTomato (1:750, goat, Sicgen), anti-Ki67 (1:500, rat, Thermo Fisher Scientific), anti-Iba1 (1:500, rabbit, Wako), anti-doublecortin (DCX) (1:500, rabbit, Cell Signaling), anti-GFAP (1:500, guinea pig, Synaptic Systems), and anti-SOX2 (1:500, rat, Invitrogen). Secondary antibodies (1:250, The Jackson Laboratory) against the respective species were used: Alexa Fluor 488 for Iba1, GFAP, and SOX2; Cy3 for tdTomato; and Alexa Fluor 647 or Cy5 for Ki67 and DCX, together with DAPI (1:1000, Thermo Fisher Scientific). Sections were mounted with a mounting medium (Immu-Mount, Epredia). Images were obtained using widefield microscopes (Axio Scan Z1, Zeiss) with excitation at 385, 475, 555, 633 nm for DAPI, Alexa Fluor 488, Cy3, and Alexa Fluro 647 or Cy5, respectively. Neonatal mouse tissue of *Gli1-Cre*^*+/−*^*Ai14*^*+/+*^ mice (shown in Fig. 2b) was analyzed 4 days after Tam (70 mg/kg). Brains were fixed in 4% PFA overnight at 4°C and transferred to 30% sucrose for 24 hours. The tissue was sectioned into 40 μm thick sections using a sliding microtome. Sections were incubated with DAPI and, after washing with TBS, mounted on glass slides. Images were obtained using widefield microscopes (Axio Scan Z1, Zeiss) with excitation at 385 nm (for DAPI) and 555 nm (for tdTomato fluorescence).

### Evaluation of laser-induced tissue damage

**Extended Data Figure 3.**
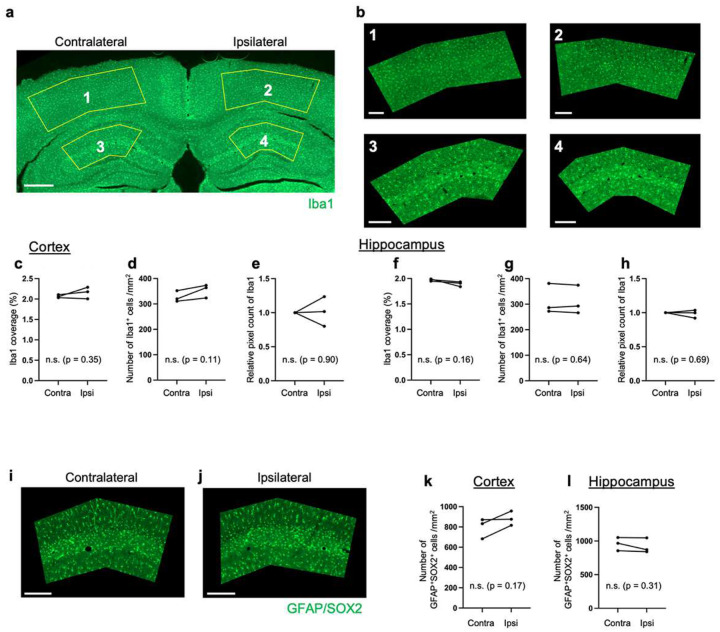
Analysis of tissue damage after chronic 3P imaging in the adult DG by immunostaining. **a**, An immunostaining image for Iba1 of a brain section after longitudinal imaging. Left: contralateral side (non-imaged) and right: ipsilateral (imaged). **b**, Close-up images of selected regions of interests (ROIs) of panel a. **c-e,** Comparison of Iba1 expression in the neocortex of the contralateral and ipsilateral hemispheres. (c) Iba1 coverage, (d) number of Iba1^+^ cells per mm^2^, and (e) relative pixel count of Iba1 in the selected ROIs in panel a. **f-h**, Iba1 expression comparison for the hippocampus area in ROIs in panel a. **i-j**, An immunostaining image for GFAP/SOX2 in the hippocampus of a brain section after longitudinal imaging. (i) Contralateral side, and (j) ipsilateral side. **k-l,** Number of GFAP^+^SOX2^+^cells per mm^2^ in the cortex (k) and the hippocampus (l). Left and right columns show the contralateral side (non-imaged) and the ipsilateral side (imaged), respectively. Scale bars represent 500 μm (a) and 200 μm (b, i, j).

**Extended Data Figure 4.**
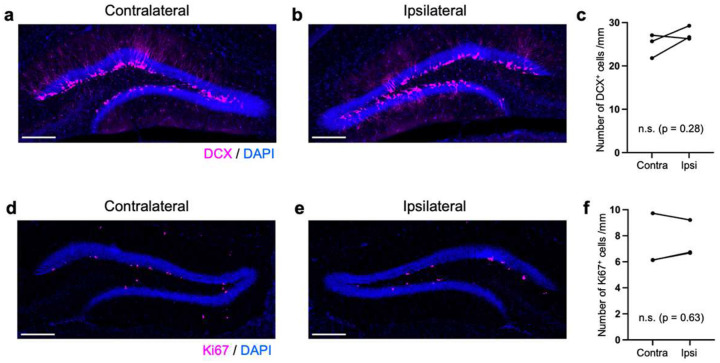
Analysis of neurogenesis after chronic 3P imaging in the adult DG by immunostaining. **a-b,** An immunostaining image for doublecortin (DCX) of a brain section after longitudinal imaging. Left: contralateral side (non-imaged) and right: ipsilateral (imaged). DCX^+^ newborn cells (magenta) in the adult DG with DAPI staining (blue). **c,** Comparison of DCX^+^ cells per mm along the subgranular zone in the contralateral and ipsilateral sides. **d-e**, An immunostaining image for Ki67 of a brain section after longitudinal imaging. Left: contralateral side (non-imaged) and right: ipsilateral (imaged). Ki67^+^ proliferating cells (magenta) in the adult DG with DAPI staining (blue). **f,** Comparison of Ki67^+^ proliferating cells per mm along the subgranular zone in the contralateral and ipsilateral sides. Scale bars represent 200 μm.

**Extended Data Figure 5.**
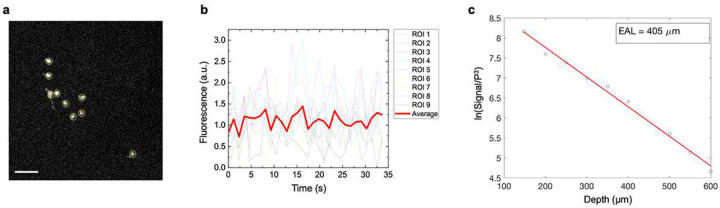
Photobleaching and attenuation length analysis. **a**, Deeptissue 3P image obtained at 1580–1610 μm depth. Yellow circles indicate ROIs used for photobleaching analysis. **b**, Fluorescence intensity traces of 9 ROIs over 35 s. Red line shows the mean of the 9 traces, indicating that photobleaching was not detected during 3P imaging. **c**, Effective attenuation length (EAL) of the adult mouse brain under the cranial window. The EAL was analyzed using the 1% brightest pixel count from the THG signals, normalized by the cube of the laser power. From the linear fit, the EAL was calculated as 405 μm, in line with the previously reported value^26^.

The effective attenuation length (EAL) was assessed by analyzing the signal decay of the THG signals acquired in the cortical layers at depths between 150–600 μm, varying the excitation laser power depending on depths. For the EAL calculation, the signal decay was plotted against the imaging depth. For the signal THG signals predominantly originating from the vasculature were used, taking the mean value of the brightest 1% of pixels in the image area. The THG signals were normalized by the cube of the laser power used for imaging and plotted against imaging depths (Extended Data Fig. 5c), similarly to the previously reported method^26^. The obtained EAL value of 405 μm was highly consistent with previously measured values at 1675 nm (365 μm)^6^ and 1700 nm (350–410 μm)^26^. To ensure imaging safety, the laser pulse energy at the focal plane was estimated. Assuming an EAL of 400 μm for the cortical and hippocampal tissues, and 150 μm for the corpus callosum (thickness: 200 μm)^26^, the total attenuation factor at the maximum imaging depth of 1600 μm was estimated to be ~4.8. This estimation indicates that the laser is attenuated by *e*^4.8^ ~ 125 times at the beam focus compared to the surface pulse energy. Given that our pulse energy under the objective was ~160–200 nJ, the pulse energy at the beam focus was calculated to be ~1.3–1.6 nJ, which is below the established laser ablation threshold^14^. Laser-induced damage was further analyzed using immunohistochemistry to quantify the activation of Iba1-expressiong microglial cells and GFAP/SOX2-expressing astrocytes, in addition to DCX-expressing newborn neurons and Ki67-expressing proliferating cells. For Iba1-labeled slices, specific ROIs were selected in the neocortex and hippocampal regions, each for contralateral (i.e., the hemisphere that was not used for longitudinal 3P imaging) and ipsilateral (i.e., the hemisphere used for longitudinal 3P imaging) (Extended Data Fig. 3a,b). Then, for each ROI, 3% thresholding was applied to generate binary images. With applying watershed and fill holes functions in ImageJ, the number of cells was counted with the Analyze Particles function of ImageJ, with a size range of 30–500 μm^2^ and a circularity of 0.1–1.0. The number of cells multiplied by the average size (μm^2^) was divided by the total area of the ROI, which gave the coverage (%) of cells in the ROI (Extended Data Fig. 3c,f). The number of cells per mm^2^ was calculated, dividing the number of cells by the total area of the ROI (Extended Data Fig. 3d,g). The mean pixel count was calculated for each ROI after subtracting the dark count (Extended Data Fig. 3e,h). Likewise, the number of cells per mm^2^ was counted for GFAP/SOX2-expressing cells (using 7% thresholding, a size range of 30–300 μm, and a circularity of 0.1–1.0) (Extended Data Fig. 3i–l). Moreover, the numbers of cells for immature neurons (DCX-expressing cells) and proliferating cells (Ki67-expressing cells) were compared in the subgranular zone (SGZ) of the contralateral and ipsilateral hemispheres (Extended Data Fig. 4a–f). For all slices containing the DG area, the length of the SGZ was measured by drawing a line across the SGZ, inner side of the granule cell layer that shows strong DAPI signals from the highly dense granule cells. Then, across the line, the numbers of cells were counted for DCX^+^ and Ki67^+^ cells, which were then averaged for contralateral and ipsilateral sides (Extended Data Fig. 4a–f).

### Statistics and reproducibility

Datasets in figures showing statistics (Fig. 1g–k and Extended Data Figs. 3c–h,k–l and 4c,f) are presented as the mean ± standard deviation. Statistical analysis was performed with GraphPad Prism (version 10.2.3). For Fig. 1g–k, each data point displays a cell. For the intact brain data from our work, the data point size (N) was 12 for Fig. 1g, 21 for Fig. 1h, 7 for Fig. 1i,j, and 51 for Fig. 1k. For the cortex-removed brain data from a previous publication^9^, N was 56 for Fig. 1g, 37 for Fig. h, 39 for Fig. 1i,j, and 543 for Fig. 1k. For Fig. 1g–k, a two-tailed unpaired t test with Welsh’s correction was applied (for comparison between the two groups). For Extended Data Figs. 3c–h,j–k and 4c,f, which compared results between contralateral and ipsilateral from the same animals, a paired t test was performed. The biological N for these comparisons was the number of mice (n = 3). Statistical significance was determined based on the P-value, with P > 0.05 defined as not significant. For longitudinal imaging, three mice with mixed sex (2 males and 1 female) were used, all within the same age group (11–14 weeks old at the start of imaging). The age range was chosen for valid comparison with previously published data^9^. The investigators were not blinded during the data acquisition (longitudinal imaging) but were blinded for the subsequent tissue analyses and quantifications.

## Supplementary Material

Captions Supplementary Videos

**Supplementary Video 1. Z-stack of three-photon imaging in the adult dentate gyrus.** A 3 × 3 × 3 voxel mean filter was applied. Maximum projection image is shown in Fig. 1d. Scale bar represents 50 μm.

**Supplementary Video 2. Time-lapse movie of three-photon imaging in the adult dentate gyrus.** Denoising by Noise2Void was applied. Selected images are shown in Fig. 1e. Scale bar represents 50 μm.

**Supplementary Video 3. Z-stack of three-photon imaging in the P7 dentate gyrus.** A 3 × 3 × 3 voxel mean filter was applied. Green: third-harmonic generation (THG), magenta: tdTomato-labeled cells. Maximum projection image is shown in Fig. 2c. Scale bar represents 100 μm.

Supplementary Files

This is a list of supplementary files associated with this preprint. Click to download.

- SupplementaryVideo1.mp4
- SupplementaryVideo2.mp4
- SupplementaryVideo3.mp4

## Extended Data
